# A New Compound Fault Feature Extraction Method Based on Multipoint Kurtosis and Variational Mode Decomposition

**DOI:** 10.3390/e20070521

**Published:** 2018-07-10

**Authors:** Wenan Cai, Zhaojian Yang, Zhijian Wang, Yiliang Wang

**Affiliations:** 1 College of Mechanical Engineering, Taiyuan University of Technology, Taiyuan 030024, China; 2 College of Mechanical and Power Engineering, North University of China, Taiyuan 030051, China

**Keywords:** multipoint kurtosis, Variational Mode Decomposition, compound fault, feature extraction

## Abstract

Due to the weak entropy of the vibration signal in the strong noise environment, it is very difficult to extract compound fault features. EMD (Empirical Mode Decomposition), EEMD (Ensemble Empirical Mode Decomposition) and LMD (Local Mean Decomposition) are widely used in compound fault feature extraction. Although they can decompose different characteristic components into each IMF (Intrinsic Mode Function), there is still serious mode mixing because of the noise. VMD (Variational Mode Decomposition) is a rigorous mathematical theory that can alleviate the mode mixing. Each characteristic component of VMD contains a unique center frequency but it is a parametric decomposition method. An improper value of K will lead to over-decomposition or under-decomposition. So, the number of decomposition levels of VMD needs an adaptive determination. The commonly used adaptive methods are particle swarm optimization and ant colony algorithm but they consume a lot of computing time. This paper proposes a compound fault feature extraction method based on Multipoint Kurtosis (MKurt)-VMD. Firstly, MED (Minimum Entropy Deconvolution) denoises the vibration signal in the strong noise environment. Secondly, multipoint kurtosis extracts the periodic multiple faults and a multi-periodic vector is further constructed to determine the number of impulse periods which determine the K value of VMD. Thirdly, the noise-reduced signal is processed by VMD and the fault features are further determined by FFT. Finally, the proposed compound fault feature extraction method can alleviate the mode mixing in comparison with EEMD. The validity of this method is further confirmed by processing the measured signal and extracting the compound fault features such as the gear spalling and the roller fault, their fault periods are 22.4 and 111.2 respectively and the corresponding frequencies are 360 Hz and 72 Hz, respectively.

## 1. Introduction

At present, there is an urgent need for state detection, feature extraction and more accurate adaptive noise reduction methods for compound fault extraction of rotating machinery. There are always weak fault characteristic components in compound faults of rotating machinery, such as pitting and micro-cracks in the gears or bearings, unbalancing of the active shaft and periodic vibrations during the rotation. When local defects happen, the compound fault features and the noise will have dynamic changes, characteristic information coupling, strong time-varying property and periodic impulse [1]. In addition, the weak fault information in compound faults will be submerged by the noise which increases the difficulty of fault feature extraction. Therefore, it is urgent to develop a reasonable and accurate adaptive fault feature extraction method.

Traditional methods have developed rapidly for single fault diagnosis. Spectrum analysis, envelope spectrum, empirical mode decomposition, ensemble empirical mode decomposition and local mean value decomposition and so forth, have shown good results in single fault diagnosis. However, the imbalanced characteristic intensity of the compound faults causes the frequency-domain processing method to be immune to weak faults [2]. In order to decompose different fault features into different time scales, Empirical Mode Decomposition (EMD) has been widely used by scholars and has achieved significant results but its mode mixing needs further processing. Ensemble Empirical Mode Decomposition (EEMD) avoids the endpoint effect of EMD but the decomposition accuracy of EEMD is influenced by the added white noise and an improper choice of the added white noise will cause over-decomposition or under-decomposition [3,4]. Local Mean Decomposition (LMD) is widely used in compound fault diagnosis but mode mixing still exists [5]. In order to decompose different time scales into different intrinsic mode functions, Variational Mode Decomposition (VMD) was proposed in 2014 [6,7,8] and has been developing rapidly in recent years. But the decomposition accuracy of VMD is affected by the penalty factor and the number of the decomposition level K. If the K value is too large or too small, over-decomposition or under-decomposition will occur [9], so the K value needs to be optimized. Considering that compound vibration fault signals contain multiple fault features and noise, it is necessary to determine the number of fault features in advance to determine the K value and then decompose the fault signals one by one. That is, when the value of K is too large, over-decomposition occurs and the white noise has been decomposed. When the K value is too small, under-decomposition occurs and results in the failure to extract some fault features. In addition, VMD is sensitive to noise [10] and the decomposition results are easily affected by the background noise. Especially in a strong noise environment, false components caused by noise are more likely to be generated, which results in misdiagnosis of faults [11,12].

For the self-determination of the K value, Yi et al. [13] used particle swarm optimization (PSO) to determine the number of decomposition level K of VMD. Zhang et al. [14] used a grasshopper optimization algorithm (GOA) to optimize VMD. Compared to the K value determined by personal experience, these optimization algorithms automatically determine the K value based on the original signal, which have good adaptability and eliminate the influence of human factors on the decomposition results. However, these algorithms need to set a large population density and the drawbacks of these parameter optimization algorithms are very obvious. There are problems such as large amount of calculation, high redundancy and low computational efficiency.

Considering that the kurtosis has nothing to do with the speed, size and load of the bearings or gears, it is sensitive to the impulse signals. The kurtosis is suitable for the diagnosis of surface micro-cracks, early damage etc. [15]. The singularity of the signal can be reflected by the kurtosis but the kurtosis cannot provide information such as the location of the fault. The multipoint kurtosis can be used to measure the period and energy of the fault feature [16]. Therefore, the number of fault periods can be determined in advance by multipoint kurtosis. According to the number of fault features, the K value in VMD is further determined adaptively, which increases the accuracy of decomposition. In recent years, Minimum Entropy Deconvolution (MED) has been widely used in fault diagnosis of gearbox [17]. MED denoises the original signal with the kurtosis maximum iterative termination condition. Therefore, MED can reduce the effect of noise on VMD and multi-point kurtosis and increase the decomposition accuracy of multipoint kurtosis. In this paper, the position of the fault has been identified in the simulation signal and the measured signal and the compound fault features of the enclosed power flow experiment table have been effectively recognized.

The article is divided into five parts, the first part is the introduction; the second part is the background and the new method; the third part is MKurt-VMD; the fourth part is Simulation signal analysis by MKurt-VMD; the fifth part is Gearbox compound fault feature extraction.

## 2. Background and New Method

### 2.1. Introduction of Variational Mode Decomposition

The VMD algorithm follows the concept of the Intrinsic Mode Function (IMF) in EMD. The central frequency and bandwidth of each IMF component are constantly updated during the iterative solution. The final decomposition results will be adaptively decomposed according to the frequency characteristics of the original signal. The constraint of the model is that the sum of these K IMFs is equal to the original signal input.

The specific construction steps of the constrained variational model are as follows:

Step 1 For the input signal x(t), through the Hilbert Transform (HT), we can get the analytic signal of each mode function $u_{k}(t)$.
Step 2 The center frequency $\omega_{k}$ of each mode function $u_{k}(t)$ is estimated and its spectrum is moved to the baseband.
Step 3 After step 2, the bandwidth is estimated through the $H^{1}$ Gauss smoothness. The final constraint variational model can be expressed by Equation (1).
  (1) $$\left\{ \begin{array}{l} \underset{\left(u_{k})(\omega_{k}\right)}{\min}\left\{\sum_{k}\|{\partial_{t}\left[\left(\sigma (t)+\frac{j}{\pi t})u_{k}(t\right)\right]e^{-j\omega_{k}t}\|}_{2}^{2}\right\}\\ s.t.\sum_{k}u_{k}=x(t) \end{array}\right.$$

In the Equation (1), $\partial_{t}$ indicates the partial derivative of *t*, σ(t) is the impulse function and $\left\{u_{k}\right\}=\left\{u_{1},\ldots ,u_{K}\right\}$ represents the K IMFs obtained by VMD of the original signal x(t), $\left\{\omega_{k}\right\}=\left\{\omega_{1},\ldots ,\omega_{K}\right\}$ represents the central frequency of each IMF component. In order to find the optimal solution to the above variational problem, the following form of Lagrange function is introduced.

(2) $$\begin{array}{l} L(\left\{u_{k}\right\},\left\{\omega_{k}\right\},\lambda )=\alpha {\sum_{k}\|\left[(\sigma (t)+\frac{j}{\pi t}\right)\times u_{k}(t)]e^{-j\omega_{k}t}\|}_{2}^{2}\\ +{\|x(t)-\sum_{k}u_{k}(t)\|}_{2}^{2}+\langle \lambda (t),x(t)-\sum_{k}u_{k}(t)\rangle \end{array}$$

In the Equation (2), λ is a Lagrange multiplier and α is a penalty factor.

Secondly, the Lagrange function of Equation (2) is transformed in time-frequency domain and the corresponding extremum solution has been carried out. The frequency domain expression of the mode function $u_{k}$ and the central frequency $\omega_{k}^{}$ can be obtained.

(3) $${\overset{\wedge }{u}}_{k}^{n+1}(\omega )=\frac{\overset{\wedge }{f}\left(\omega \right)-\sum_{i\neq k}{\overset{\wedge }{u}}_{i}(\omega )+\frac{\overset{\wedge }{\lambda }(\omega )}{2}}{1+2\alpha {\left(\omega -\omega_{k}\right)}^{2}}$$

(4) $$\omega_{k}^{n+1}=\frac{{\int_{0}^{\infty }\omega \left|{\overset{\wedge }{u}}_{k}(\omega )\right|}^{2}d\omega }{{\int_{0}^{\infty }\left|{\overset{\wedge }{u}}_{k}(\omega )\right|}^{2}d\omega }$$

Finally, the optimal solution of the constrained variational model is solved by using the alternate direction method of multipliers (ADMM) and the original signal x(t) has been decomposed into K IMFs.

The specific steps of the algorithm are as follows.

Step 1 Initialize the parameters, set $\left\{{\overset{\wedge }{u}}_{k}^{1}\right\}$, $\left\{{\overset{\wedge }{\omega }}_{k}^{1}\right\}$, ${\overset{\wedge }{\lambda }}^{1}$ and n to 0.
Step 2 Update ${\overset{\wedge }{u}}_{k}^{}$ and ${\overset{\wedge }{\omega }}_{k}^{}$ according to Equations (3) and (4).
Step 3 Update the value of ${\overset{\wedge }{\lambda }}^{n+1}$ according to the Equation ${\overset{\wedge }{\lambda }}^{n+1}(\omega )={\overset{\wedge }{\lambda }}^{n}(\omega )+\tau (\overset{\wedge }{f}(\omega )-\sum_{k}{\overset{\wedge }{u}}_{k}^{n+1}(\omega ))$. τ is time-step of the dual ascent
Step 4 If the inequality $\frac{\sum_{k}{\left\|{\overset{\wedge }{u}}_{k}^{n+1}-{\overset{\wedge }{u}}_{k}^{n}\right\|}_{2}^{2}}{\left\|{\overset{\wedge }{u}}_{k}^{n}\right\|}<\varepsilon$ has been satisfied, the iteration stops and the loop exits. Otherwise, return to step 2. Finally, K intrinsic mode functions are obtained.

### 2.2. Introduction of MKurt

In 2016, McDonald and Qing [16] proposed a multi-pulse target identification algorithm based on Multi-D-Norm, which is mainly applied to the fault feature extraction of periodic impulses and further introduced the concept of multipoint optimized minimum entropy deconvolution adjusted (MOMEDA).

(5) $$\mathrm{Multi}\;D\text{-}\mathrm{Norm}\;=\;\mathrm{MDN}(\vec{y},\vec{t})=\frac{1}{\left\|\vec{t}\right\|}\frac{{\vec{t}}^{T}\vec{y}}{\left\|\vec{y}\right\|}$$

(6) $$\mathrm{MOMEDA}:\;\max\;\mathrm{MDN}(\vec{y},\vec{t})=\underset{\vec{f}}{\max}\frac{{\vec{t}}^{T}\vec{y}}{\left\|\vec{y}\right\|}$$

Among them, the target vector $\vec{t}$ is a constant vector that determines the impulse position and the weight. The normalized 1 is to represent the best target solution. The periods of different fault signatures at the same sampling frequency can also be identified, thus the target vector $\vec{t}$ can be used for impulse signal separation and position determination.

The filer of MOMEDA and output solution can be simply summarized in Equation (7). For a detailed calculation, see [16]:

(7) $$\vec{f}={\left(X_{0}X_{0}^{T}\right)}^{-1}X_{0}\vec{t}$$

(8) $$X_{0}={\left[\begin{array}{cccccc} x_{L} & x_{L+1} & x_{L+2} & \ldots & \ldots & x_{N}\\ x_{L-1} & x_{L} & x_{L+1} & \cdots & \cdots & x_{N-1}\\ x_{L-2} & x_{L-1} & x_{L} & \cdots & \cdots & x_{N-2}\\ \vdots & \vdots & \vdots & \ddots & \cdots & \vdots \\ x_{1} & x_{2} & x_{3} & \cdots & \cdots & x_{N-L+1} \end{array}\right]}_{L\;by\;N-L+1}$$

(9) $$\vec{y}=X_{0}^{T}\vec{f}$$

The target vector $\vec{t}$ has the same length of (*N* − *L* + 1). The target vector represents the position and the weight of the deconvolution pulse in the output, the position of the pulse has been controlled. MOMEDA obtains the optimal solution through a non-iterative period, which provides the basis of the periodic fault diagnosis. MOMEDA can also perform the following calculations on M consecutive target vectors, where Equations (7) and (9) will be transformed to Equations (10) and (11).

(10) $$F=\left[\begin{array}{cccc} {\vec{f}}_{1} & {\vec{f}}_{2} & \ldots & {\vec{f}}_{M} \end{array}\right]={\left(X_{0}X_{0}^{T}\right)}^{-1}X_{0}\left[\begin{array}{cccc} {\vec{t}}_{1} & {\vec{t}}_{2} & \ldots & {\vec{t}}_{M} \end{array}\right]$$

(11) $$Y=\left[\begin{array}{cccc} {\vec{y}}_{1} & {\vec{y}}_{2} & \ldots & {\vec{y}}_{M} \end{array}\right]\vec{y}=X_{0}^{T}F$$

However, the position for solving the deconvolution is unique by using MOMEDA. For a single periodic impulse, it is not necessary to consider whether the period is an integer or the length of the filter has influence on the noise reduction. A periodic impulse will appear within the entire sampling interval. However, when there are multiple periodic faults, the number of target vector $\vec{t}$ will increase. The compound fault tracking effect of MOMEDA is not good and MOMEDA cannot identify multiple periodic impulses.

In order to extract compound fault features accurately, multipoint kurtosis is introduced as a metric. First introduce a normalized factor *K* and the multipoint kurtosis is defined as:

(12) $$\mathrm{MKurt}\left(\vec{y},\vec{t}\right)=k\frac{\sum_{n=1}^{N-L}{\left(t_{n}y_{n}\right)}^{4}}{{\left(\sum_{n=1}^{N-L}y_{n}^{2}\right)}^{2}}$$

When the output $\vec{y}$ is the same as target vector $\vec{t}$, the MKurt is normalized

(13) $$l=k\frac{\sum_{n=1}^{N-L}{\left(t_{n}^{2}\right)}^{4}}{{\left(\sum_{n=1}^{N-L}t_{n}^{2}\right)}^{2}}$$

Solve the normalization factor

(14) $$k=\frac{{\left(\sum_{n=1}^{N-L}t_{n}^{2}\right)}^{2}}{\sum_{n=1}^{N-L}t_{n}^{8}}$$

The final standardized multipoint kurtosis is defined as

(15) $$\mathrm{MKurt}\;=\;\frac{{\left(\sum_{n=1}^{N-L}t_{n}^{2}\right)}^{2}}{\sum_{n=1}^{N-L}t_{n}^{8}}\frac{\sum_{n=1}^{N-L}{\left(t_{n}y_{n}\right)}^{4}}{{\left(\sum_{n=1}^{N-L}y_{n}^{2}\right)}^{2}}$$

The target vector allows a single position impulse to be expanded to multiple pulses and to be further normalized. When multiple faults coexist, there are different vibration periods for each rotation of rotating machinery and there are multiple target vectors. Therefore, multiple peaks appear in the multiple kurtosis. The peak corresponds to an impulse period, the integral multiple of the period or half of the period. If multiple faults coexist, multiple peaks will appear in the low sampling interval and the peaks are dense, making the size of the period difficult to determine. Therefore, it is necessary to determine the periodic vector and enumerate the different periodic factors together with multiples to further determine the number of periodic pulses in the compound signal. Taking into account that the peak of the main period is not the largest, using multiple kurtosis alone to determine the frequency of the impulse signal will lead to misdiagnosis. The multiple kurtosis is used to determine the number of periods, then other adaptive methods are used to decompose and extract the fault features.

In addition, the multipoint kurtosis will be buried by the noise, so the filter needs a prior determination in order to highlight the period of the impulse in a strong noise environment. The multipoint kurtosis of the simulation signal will be analyzed in Section 3. 

## 3. Introduction of MKurt-VMD method

The multipoint kurtosis can adaptively determine the multiple fault impulses but it has the following features:

1. The multipoint kurtosis searches for the target vector in noisy environment without target,
2. There are many spectral peaks in multipoint kurtosis, which are not only at integer multiple periods but also at half, one-third or quarter multiples of the periods.
3. The main period cannot be determined only by the multipoint kurtosis, because the peaks in the main period are not the largest.

**Figure 1:**
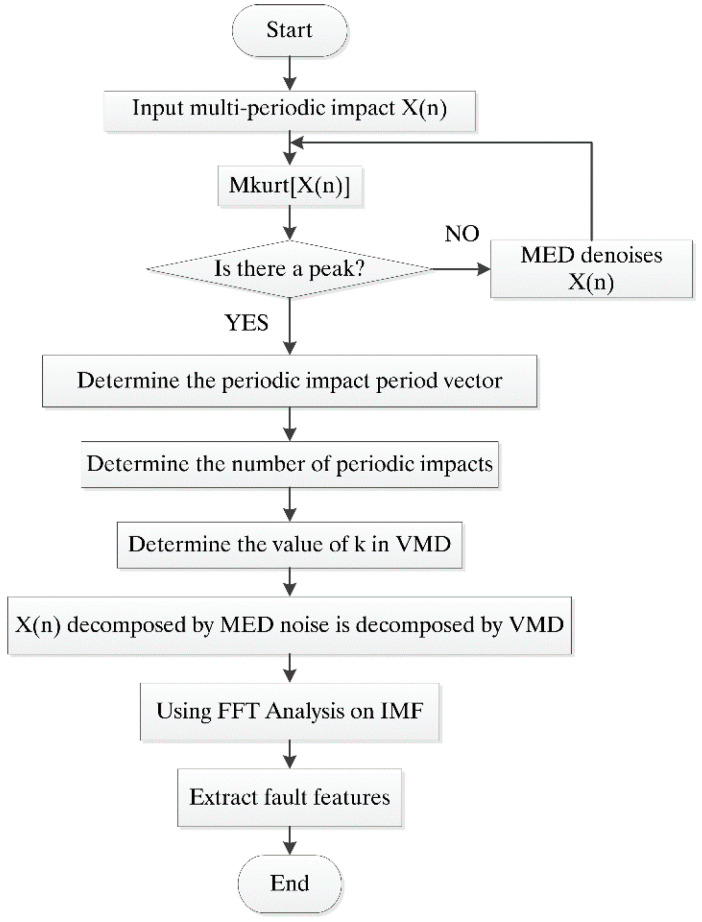
The flowchart of Multipoint Kurtosis Variational Mode Decomposition (MKurt-VMD).

In order to extract the number of fault periods with multiple fault characteristics accurately, the signal can be denoised by MED in a strong noise environment. Under the premise of determining penalty factor, the K value in VMD is further set by determining the number of periods in advance. Considering that there is a large amount of noise in the original signal, the original signal can be denoised by MED before decomposition to further improve the signal-to-noise ratio of the original signal. VMD adaptively decomposes the original signal into K IMFs from low frequency to high frequency and K frequencies are arranged in order from low frequency to high frequency. The flowchart of MKurt-VMD is in Figure 1. Specific steps of MKurt-VMD are as follows:

(1) The multipoint kurtosis of the original signal is calculated and the multipoint kurtosis spectrum is determined. The number of sampling points corresponding to the peak is listed according to the spectrum peak,
(2) If there is no peak in the vibration signal, the peak has been polluted by the noise,
(3) Perform MED on the original signal and then continue to perform step 1,
(4) List the sampling points corresponding to the peaks one by one and search for sampling points with multiple relationships, finally determine the number of periods,
(5) Determine the number of decomposition level K in VMD,
(6) Decompose the original signal by VMD,
(7) Solve the spectrum of each IMF,
(8) Determine the fault characteristics.

## 4. Simulation Signal Analysis by MKurt-VMD method

**Figure 2:**
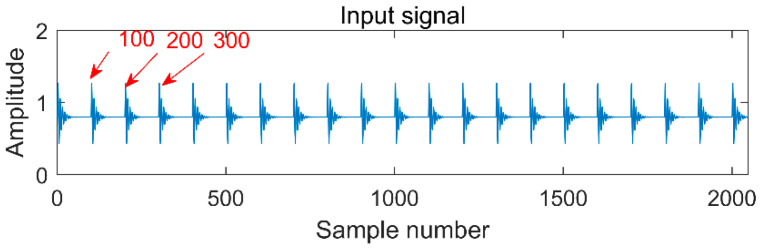
Single impulse signal.

**Figure 3:**
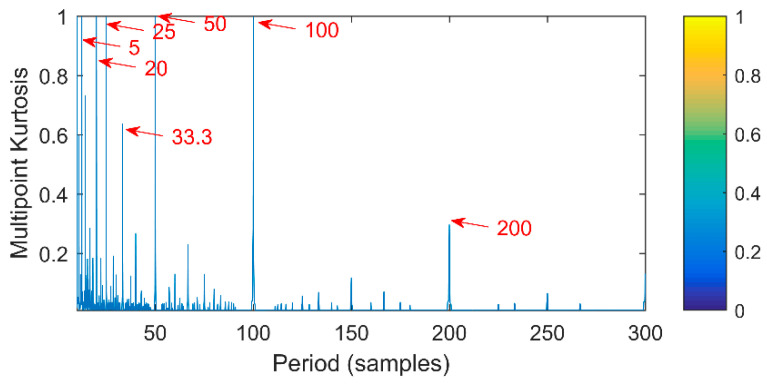
Multipoint kurtosis spectrum of the single impulse signal.

First of all, verify the performance of multipoint kurtosis. A simulation signal shown in Figure 2 is given, the sampling frequency is 2000 Hz, the number of sampling points is 2048 and the vibration period is *T* = 100. Since there is no noise, its amplitude and frequency characteristics are obvious. The vibration frequency is 20 Hz. The multipoint kurtosis spectrum is shown in Figure 3. There are many peaks in the entire interval and the density of peaks turns to be sparse with the increase of sampling points. This is due to the multipoint kurtosis appears the peaks of the vibration period and the integral multiple of the period during the searching process. The period corresponding to the peaks is counted and a period vector is obtained. These periods are [5, 20, 25, 33.3, 50, 100, 200] corresponding to [T/20, T/5, T/4, T/3, T/2, T, 2T] respectively. We know that vector represents periodic impulses. However, the corresponding period of the main peak cannot be obtained under a priori uncertainty. After observing the amplitude of each peak, it is obvious that the corresponding amplitudes at T/20, T/5, T/4 and T/2 are the same and the corresponding peak of 2*T* becomes smaller. The location of the main peak cannot be determined. A large number of main peaks will lead to misdiagnosis unless there is a priori knowledge of the impulse periods. When we simply determine the periodic impulse from the multiple kurtosis, the only information we can get is that there are indeed impulses in the vibration signal. The period of the vibration signal has not yet been solved.

**Figure 4:**
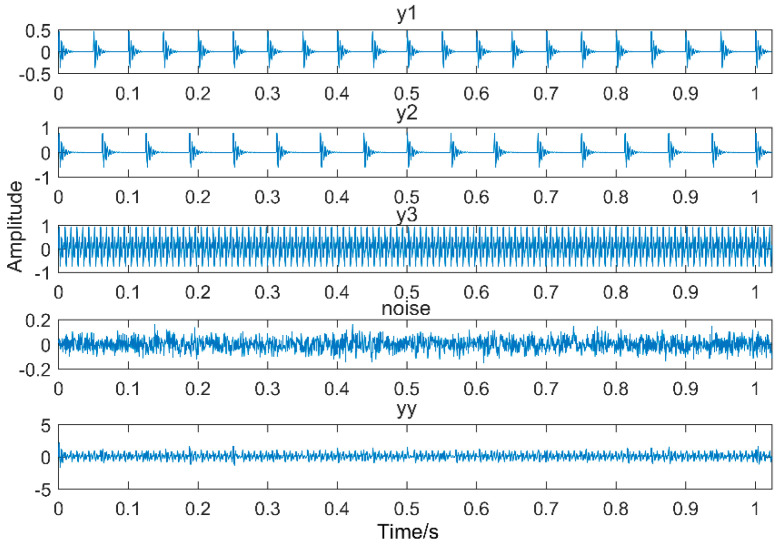
Time-domain waveforms of the multi-impulse signal.

**Figure 5:**
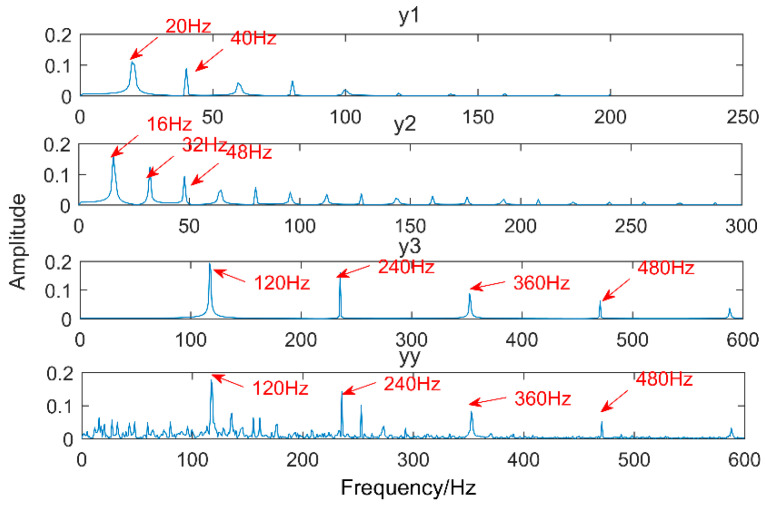
Envelope spectrum of the multi-impulse signal.

**Figure 6:**
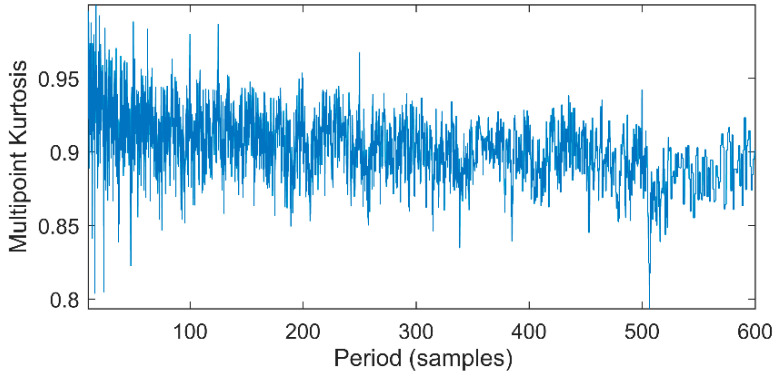
Multipoint kurtosis spectrum of the multiple impulse signal.

**Figure 7:**
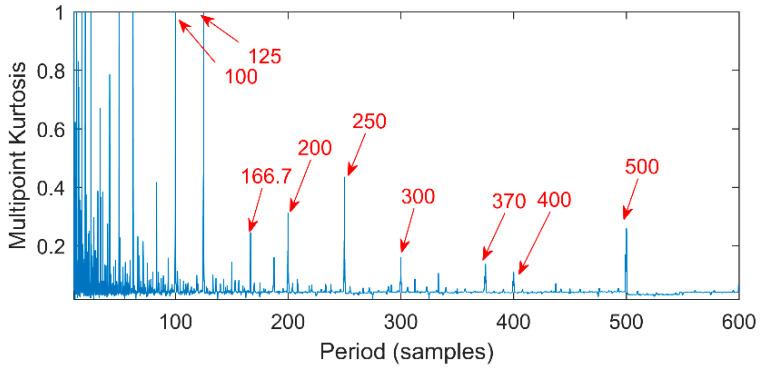
Multipoint kurtosis spectrum with interval of [10, 600].

**Figure 8:**
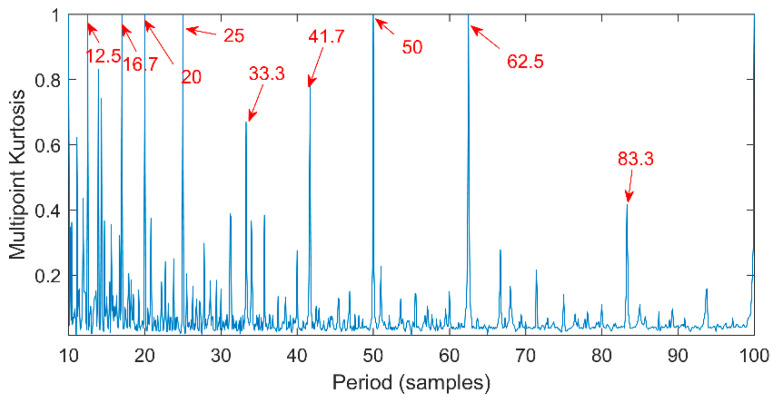
Multipoint kurtosis spectrum with interval of [10, 100].

Multipoint kurtosis analysis is now performed on a multi-impulse signal. The simulation signal has three impulse signals and one noise signal. Their frequencies are 20 Hz, 16 Hz, 120 Hz and the sampling frequency is 2000 Hz and sampling points are 2048. Their corresponding periods are *T*1 = 100, *T*2 = 125 and *T*3 = 16.6 corresponding to the simulation signal. The time domain is shown in Figure 4. The envelope spectra corresponding to y1, y2, y3 and yy are shown in Figure 5. The first three frequencies are 20 Hz, 16 Hz and 120 Hz respectively but the frequency in the synthesized signal yy is only 120 Hz. The three frequencies need to be identified simultaneously and further decomposition of the original signal is required. The multipoint kurtosis of the simulation signal is shown in Figure 6. Although there are a few peaks, the noise has distorted the true characteristics of the signal, so the multipoint kurtosis is not immune in a strong noise environment. Denoise the simulation signal by MED with the purpose of improving the signal-to-noise-ratio together with the energy of the impulse vibration. The multipoint kurtosis of the denoised signal is shown in Figure 7 and Figure 8. The search interval in Figure 7 is [10, 600] and Figure 8 is obtained in order to refine the interval [10, 100]. From the figures, we know that there is a large number of spectral peaks in the low period range and the mean values are not much different and the multipoint kurtosis is a measure of the periodic existence. The specific analysis is as follows: The periods corresponding to the two peaks can be divided into three types from low to high: [12, 5, 62.5, 125, 250, 500] = [T2/10, T2/2, T2, 2T2, 4T2], [20, 25, 50, 100, 200, 300, 400] = [T1/5, T1/4, T1/2, T1, 2T1, 3 T1, 4 T1] and [16.7, 33.3, 41.7, 66.7, 83.3, 166.7] = [T3, 2T3, 2.5T3, 4T3, 5T3, 10T3]. The three-period vectors represent three impulse periods and each vector represents a type of fault. It only shows that the original signal contains three different periodic impulses but the main frequency and the main period still remain unknown.

**Figure 9:**
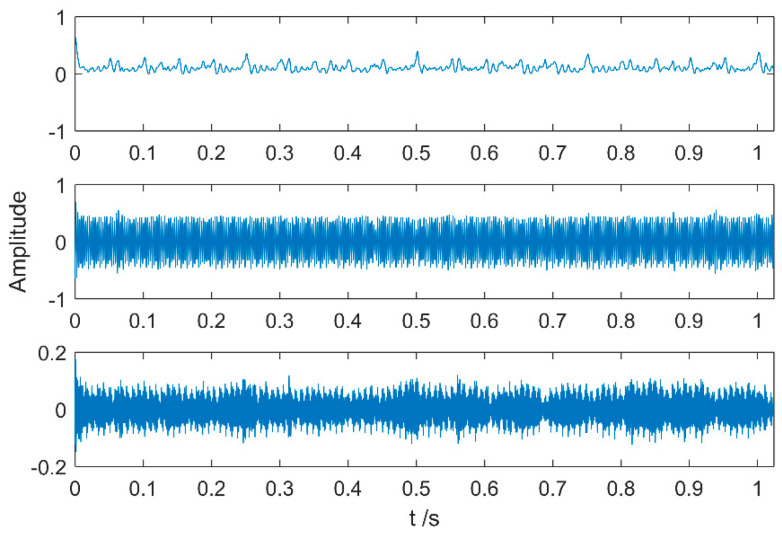
VMD results of the simulation signal at K = 3.

**Figure 10:**
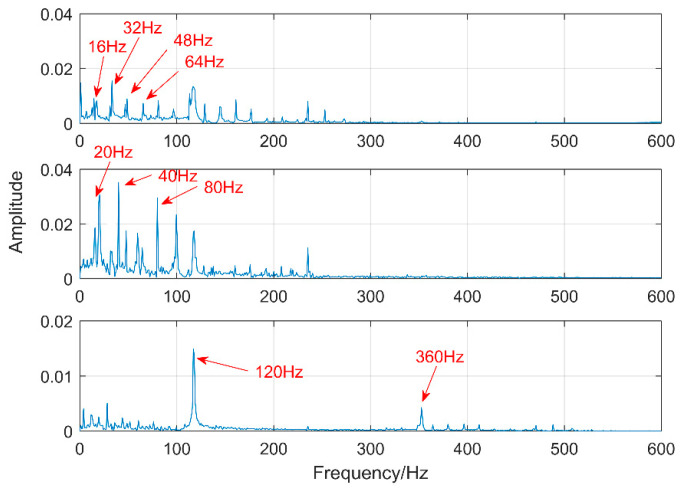
Envelope analysis of VMD of the simulation signal at K = 3.

According to the multipoint kurtosis spectrum, the number of periodic impulses in the simulation signal is determined to be three. At this time, K equals 3 and the optimized VMD decomposes the denoised simulation signal. The results are shown in Figure 9, although the three intrinsic mode functions are still polluted by a little noise. The results of processing the three IMFs through the envelope spectrum are shown in Figure 10. The three decomposed intrinsic mode functions are arranged from low-frequency to high-frequency in turn and each level contains only a time scale. The frequencies of the three levels are 16 Hz, 20 Hz and 120 Hz respectively. There is no over-decomposition in the results and there is no mode mixing.

**Figure 11:**
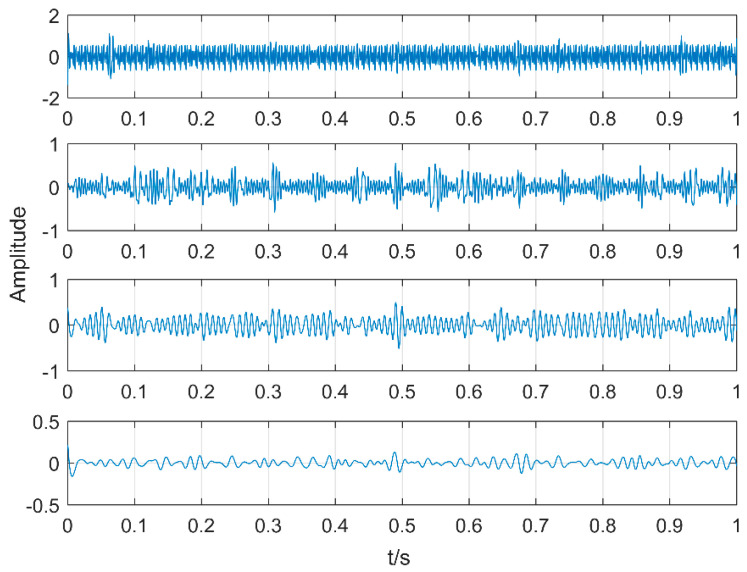
The first four levels of Intrinsic Mode Functions (IMFs) decomposed by Ensemble Empirical Mode Decomposition (EEMD).

**Figure 12:**
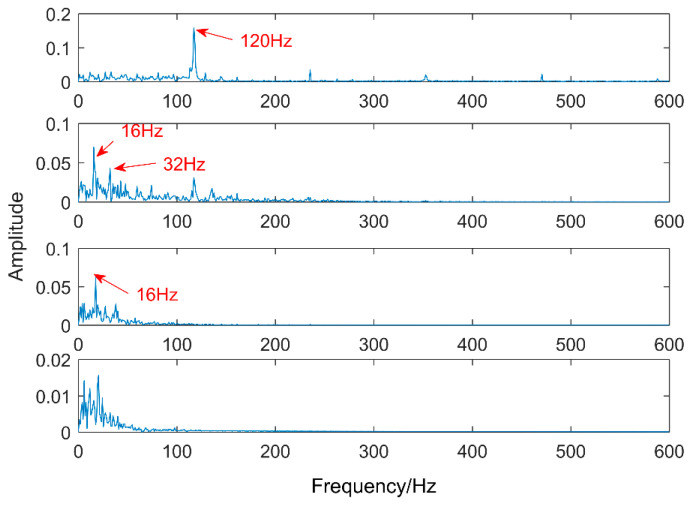
Envelope analysis of the first four IMFs.

Comparing EEMD with the proposed methods, EEMD is an adaptive noise reduction method but it is also a parametric method. The two parameters of EEMD are the amplitude of the added white noise and the added times. The amplitude of added white noise is 0.2 and the number of added times is 100 by experience. After decomposing, the first four levels of the intrinsic mode functions which have the strongest correlation with the original signal are taken and the results are shown in Figure 11. There are obvious periodic oscillations in these four levels. The corresponding envelope diagram is shown in Figure 12. The low frequencies are decomposed into four intrinsic mode functions one by one. There is serious mode mixing in the second and third levels and 20 Hz has not been extracted. The decomposition results are not as good as the method proposed in this article.

**Figure 13:**
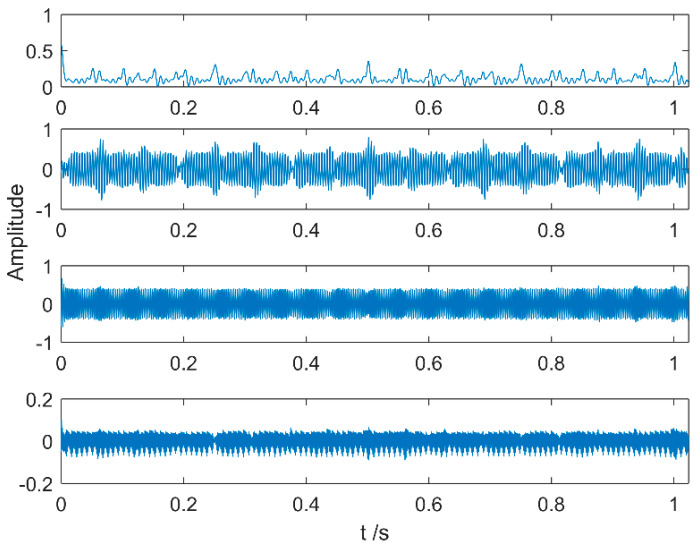
VMD results of the simulation signal at K = 4.

**Figure 14:**
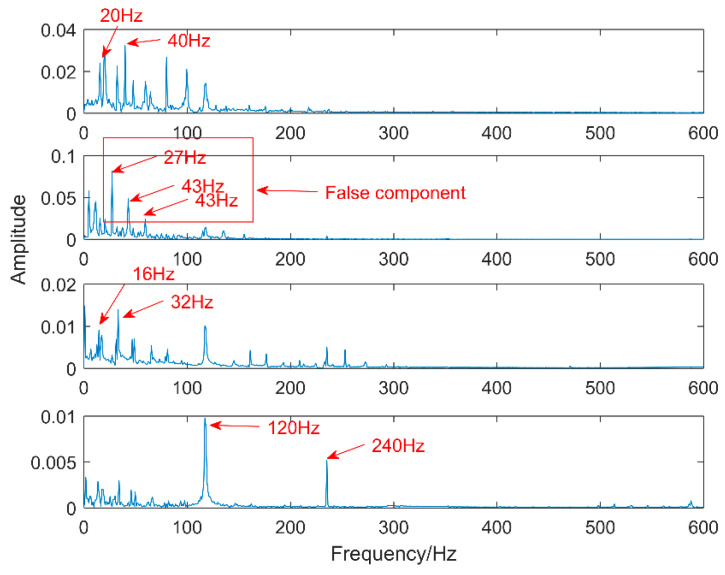
Envelope analysis of VMD of the simulation signal at K = 4.

When K = 4, the original signal is decomposed by VMD and the results are shown in Figure 13 and Figure 14. According to the envelope spectrum, it can be found that 20 Hz, 10 Hz and 120 Hz have been extracted but there is over-decomposition. The corresponding frequency of the second level does not belong to the center frequency in the simulation signal, so when the K value is too large, it may easily lead to misdiagnosis.

## 5. Gearbox Compound Fault Feature Extraction

**Figure 15:**
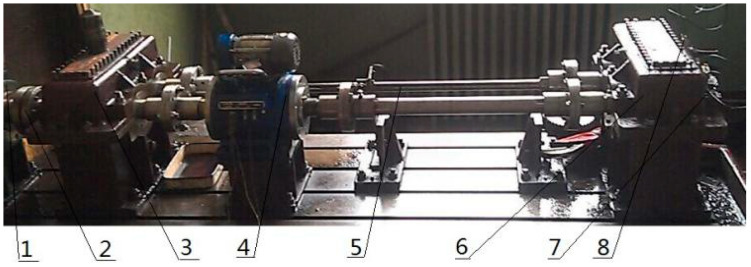
The enclosed power flow experiment table. 1—Speed-adjustable motor, 2—Coupling, 3—Accompanied gearbox, 4—Speed reversing instrument, 5—Torsion bar, 6—Test gearbox, 7—Three-way acceleration sensor 1#, 8—Three-way acceleration sensor 2#.

The test bench designed in this paper is a closed-type power flow experimental rig, which is loaded by the internal force generated by the torsion bar. The speed is adjusted by an electromagnetic speed-regulated asynchronous motor, ranging from 120 r/min to 1200 r/min. Under different loads, the vibration signal of the gear under normal and pitting conditions is measured. This chapter mainly uses compound fault as an example to verify the feasibility of the above method. The enclosed power flow experimental rig is shown in Figure 15. It includes a speed-adjustable motor, coupling, an accompanied gearbox, a speed reversing instrument, torsion bar, test gearbox and a three-way acceleration sensor. The transmission ratio is 1:1 and the half-tooth meshing (the number of teeth is 18) is adopted, the rotation speed is 1200 r/min, the rotation frequency is 20 Hz, the fault frequency of the roller is 72 Hz, the meshing frequency is 360 Hz and the sampling frequency is 8000 Hz. There are two faults in this experiment: gear spalling and the roller fault. The load torque is 1000 Nm and the test bearing model is 32212. The gear peeling is generated by the gear fatigue test and the outer ring fault is artificially implanted by the electric discharge machining method.

**Figure 16:**
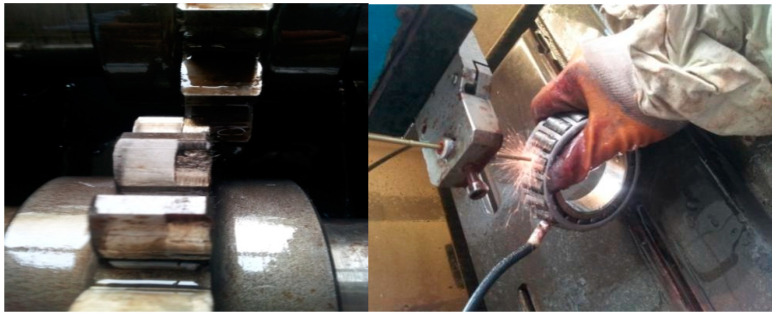
Roller fault of tapered roller bearings and gear fault.

The sensor is YD77SA three-way acceleration sensor (the sensitivity is 0.01 V/ms^2^) and the number of samples is 2048. Through a simple calculation, the roller fault period is *T*1 = 400 (sample point) and the gear meshing period is *T*2 = 22.4. Fault types are shown in Figure 16.

**Figure 17:**
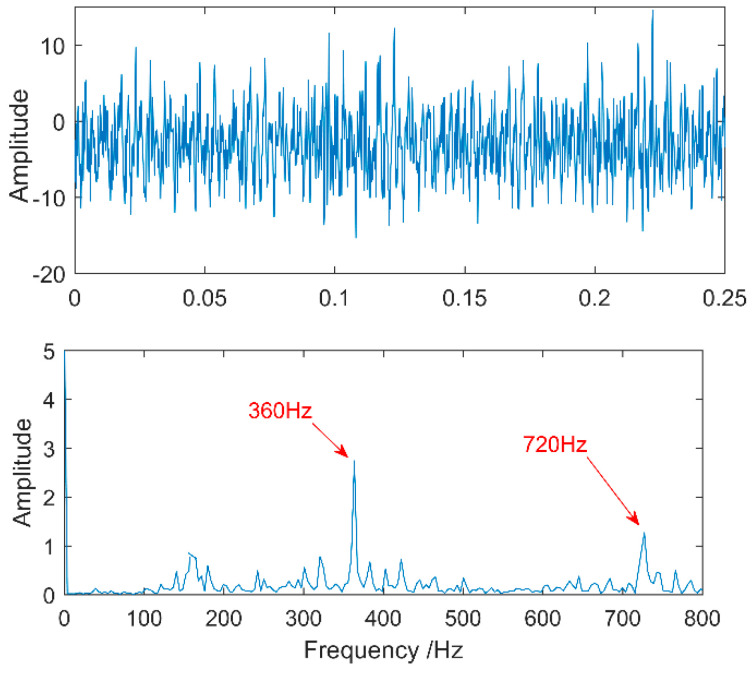
Time-domain waveform of spalling gear.

**Figure 18:**
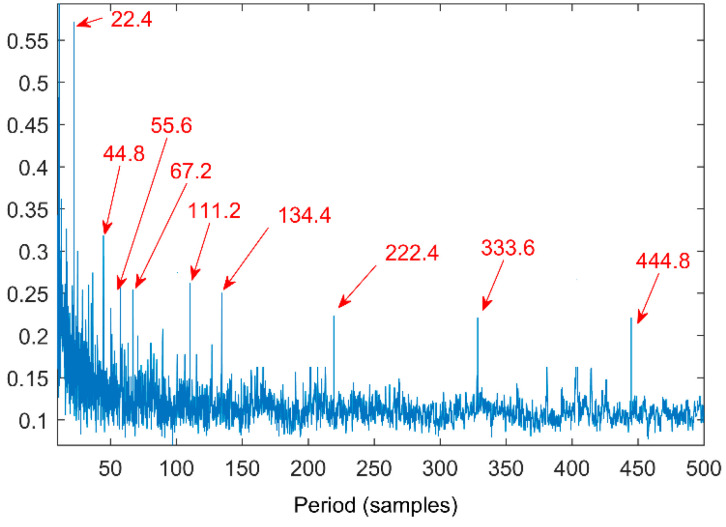
Multipoint kurtosis spectrum of fault gearbox.

**Figure 19:**
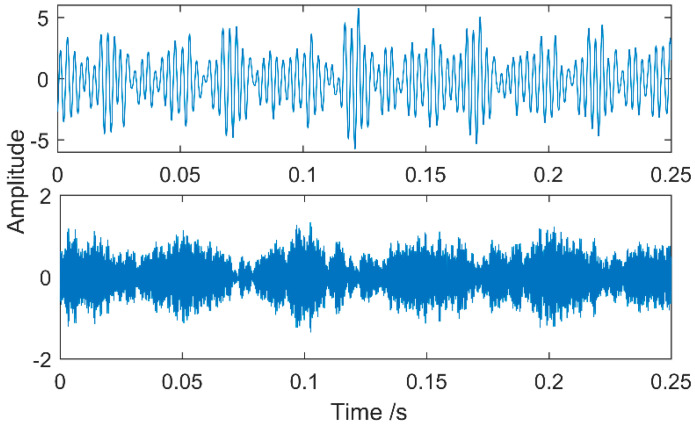
VMD results of the vibration signal when K = 2.

**Figure 20:**
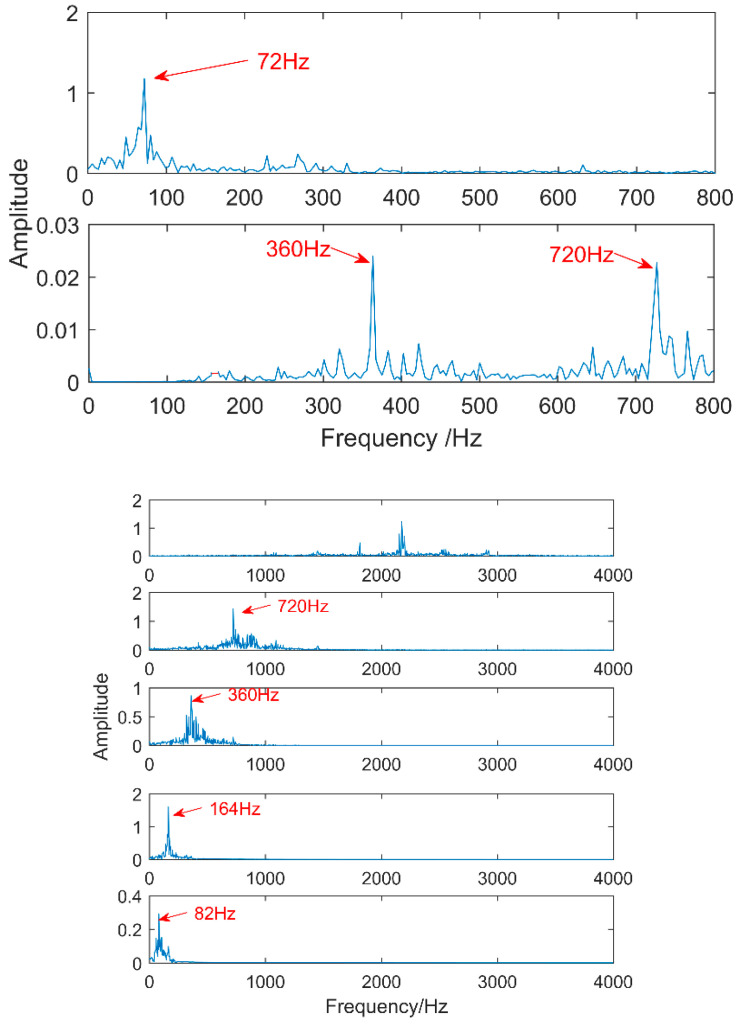
VMD results of the vibration signal and FFT when K = 2.

**Figure 21:**
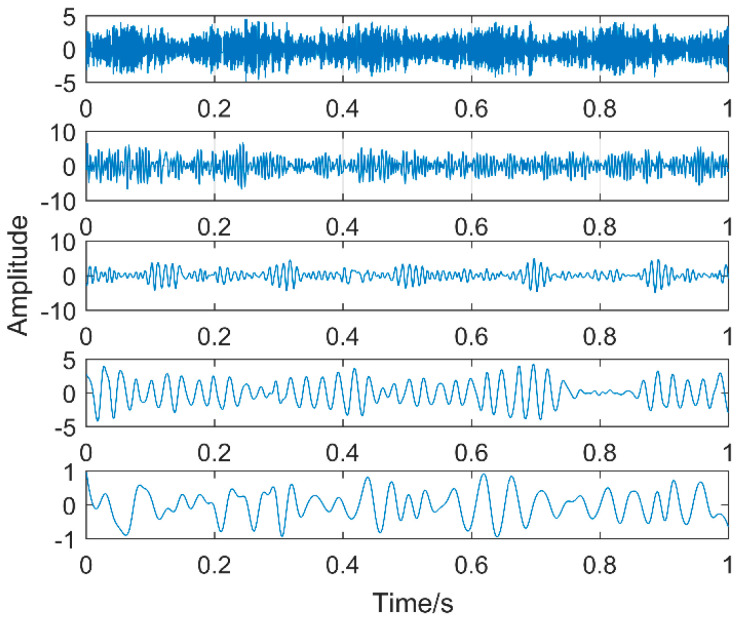
Vibration signal decomposed by EEMD and its spectrum.

The time waveform of the compound fault vibration signal is shown in Figure 17. With the increase of the load, the noise increases gradually. Obvious impulses in the figure and the gear fault can be preliminarily determined through spectrum analysis. The multipoint kurtosis spectrum analysis of the fault data results is shown in Figure 18. The two periodic are 22.4 and 111.2. [22.4, 44.8, 67.2, 134.4] = [T2, 2T2, 3T2, 6T2] and [55.6, 111.2, 222.4, 333.6, 444.8] = [T1/2, T1, 2T1, 3T1, 4T1], which represent the meshing period of the gear and the fault period of the roller respectively and there are two primary faults. In order to successfully extract these two fault characteristics, the fault signal is denoised by MED and then decomposed by VMD. The K value is 2 corresponding to Figure 19 and Figure 20. The three frequencies in Figure 21 are 72 Hz, 180 Hz and 360 Hz corresponding to the roller fault frequency, gear meshing frequency and its double frequency. The fault location has been preliminarily determined and the fault characteristics have been extracted. In order to further analyze the results of the original signal decomposed by EEMD, the white noise amplitude is 0.2 and the number of added times is 100. Taking the first five IMFs which have the strongest correlation with the original signal, the corresponding results are shown in Figure 21. The second and third level fault characteristics correspond to the fault characteristics of the gear and the first, the fourth and the fifth levels is not related to the original signal. The decomposition accuracy of EEMD is affected by the noise and the added white noise. Improper selection of white noise is likely to lead to misdiagnosis. At the same time, the effectiveness of the proposed method in this article has been demonstrated.

## 6. Conclusions

(1) The decomposition level of VMD is generally determined by experience. In engineering applications, K too large or too small will lead to over-decomposition or under-decomposition.
(2) MKurt can solve the compound fault impulse periods. A large number of spectral peaks appear in the multipoint kurtosis spectrum and this method is immune in noisy environments. According to this feature, the number of VMD decomposition level can be determined adaptively.
(3) Both the VMD and the multipoint kurtosis are affected by the noise. In a strong noise environment, MED can be used as the prefilter to increase the signal-to-noise ratio of the signal. The proposed MKurt-VMD method can improve the decomposition accuracy. The method proposed in the article determines the fault cycle of gears and rollers, which are 22.4 and 111.2 respectively and the corresponding frequencies are 360 Hz and 72 Hz, respectively. This paper verifies the reliability of the method and verifies the certain reference value of this method in engineering applications in comparison to EEMD. Of course, for non-periodic shock signals, the proposed method has limitations.
